# Altered EEG spectral power during rest and cognitive performance: a comparison of preterm-born adolescents to adolescents with ADHD

**DOI:** 10.1007/s00787-017-1010-2

**Published:** 2017-06-02

**Authors:** Anna-Sophie Rommel, Sarah-Naomi James, Gráinne McLoughlin, Daniel Brandeis, Tobias Banaschewski, Philip Asherson, Jonna Kuntsi

**Affiliations:** 1 MRC Social, Genetic and Developmental Psychiatry Centre, Institute of Psychiatry, Psychology and Neuroscience, King’s College London, DeCrespigny Park, London, SE5 8AF UK; 2 Medical Research Council Unit for Lifelong Health and Ageing at University College London, London, UK; 3 Department of Child and Adolescent Psychiatry and Psychotherapy, Central Institute of Mental Health, Medical Faculty Mannheim/Heidelberg University, Mannheim, Germany; 4 Department of Child and Adolescent Psychiatry and Psychotherapy, Psychiatric Hospital, University of Zurich, Zurich, Switzerland; 5 Center for Integrative Human Physiology, University of Zurich, Zurich, Switzerland; 6 Neuroscience Center Zurich, University of Zurich, Zurich, Switzerland

**Keywords:** ADHD, Preterm birth, Quantitative EEG, Neurocognitive impairment, Delta power

## Abstract

Preterm birth has been associated with an increased risk for ADHD-like behavioural symptoms and cognitive impairments. However, direct comparisons across ADHD and preterm-born samples on neurophysiological measures are limited. The aim of this analysis was to test whether quantitative EEG (QEEG) measures identify differences or similarities in preterm-born adolescents, compared to term-born adolescents with and without ADHD, during resting-state and cognitive task conditions. We directly compared QEEG activity between 186 preterm-born adolescents, 69 term-born adolescents with ADHD and 135 term-born control adolescents during an eyes-open resting-state condition (EO), which previously discriminated between the adolescents with ADHD and controls, and during a cued continuous performance task (CPT-OX). Absolute delta power was the only frequency range to demonstrate a significant group-by-condition interaction. The preterm group, like the ADHD group, displayed significantly higher delta power during EO, compared to the control group. In line with these findings, parent-rated ADHD symptoms in the preterm group were significantly correlated with delta power during rest. While the preterm and control groups did not differ with regard to absolute delta power during CPT-OX, the ADHD group showed significantly higher absolute delta power compared to both groups. Our results provide evidence for overlapping excess in the absolute delta range in preterm-born adolescents and term-born adolescents with ADHD during rest. During CPT-OX, preterm-born adolescents resembled controls. Increased delta power during rest may be a potential general marker of brain trauma, pathology or neurotransmitter disturbances.

**Electronic supplementary material:**

The online version of this article (doi:10.1007/s00787-017-1010-2) contains supplementary material, which is available to authorized users.

## Introduction

Preterm births, denoting births before 37 completed weeks of gestation [1], constitute 8.6% of births in the developed world [2]. Although the survival rates for individuals born preterm have increased greatly over the past decades [3], preterm birth is a pre- and peri-natal trauma that increases the risk of adverse long-term outcomes [4], including the risk for attention-deficit/hyperactivity disorder (ADHD) possibly because the late third trimester (32–40 weeks’ gestation) serves as a critical period in laying the foundation of brain networks [5]. One meta-analysis demonstrated that preterm-born children (*n* = 1556) were at heightened risk (relative risk, RR 2.64) of developing ADHD relative to controls (*n* = 1720) [6]. A population-based study of Norwegian adults further reported a 1.3- and fivefold increased risk for ADHD in adults born preterm (<37 weeks) and extremely preterm (<28 weeks), respectively [7].

A cognitive profile that resembles that of individuals with ADHD, including impairments in attention and inhibitory control, is also frequently associated with preterm birth [8, 9]. Yet, it is unclear whether the symptoms and cognitive impairments seen in preterm-born individuals are identical to those associated with ADHD or whether they are part of more wide-ranging impairments.

Quantitative electroencephalography (QEEG) allows investigation of covert processes due to its sensitivity to subtle changes in the power of oscillatory activity and its high temporal resolution [10]. QEEG is, therefore, a useful tool to examine and compare the neurocognitive profiles of preterm-born individuals and individuals with ADHD. Electrophysiological recordings are quantified and conventionally described in the following frequency bands: delta (0.5–3.5 Hz), theta (3.5–7.5 Hz), alpha (7.5–12.5 Hz), beta (12.5–30 Hz) and gamma (>30 Hz) [11–14]. Traditionally, elevated power in slow (delta and theta) and reduced power in fast (mainly beta) frequency bands have been reported for individuals with ADHD [15–18] during resting-state conditions. However, a number of recent studies have not replicated these findings [19–22]. EEG spectral power in all frequency bands is affected by age [23–25]. In neurotypical individuals, for instance, absolute EEG spectral power in all frequency bands decreases from childhood into adolescence, whereas relative EEG spectral power increases in the fast bands and decreases in the slow bands [23, 24]. In individuals with ADHD, age has also been shown to influence EEG spectral power [21, 25, 26]. Yet, research on the development of EEG spectral power in individuals with ADHD compared to age-matched controls has yielded inconsistent results. One study found elevated theta power in ADHD throughout the lifespan [15], whereas other studies showed atypical developmental trajectories in individuals with ADHD during late childhood [21] or adulthood [19].

Although several EEG studies have examined the neurophysiology of preterm-born infants in neonatal intensive care units [27] and in the postnatal period [28], few EEG studies have been conducted in children, adolescents and adults born preterm. One study that investigated QEEG in children with educational problems during an eyes-closed (EC) resting condition reported increased theta (3.6–5 Hz), beta (20.1–30 Hz) and gamma (30.1–40.2 Hz) power in preterm-born children, compared to term-born controls with education problems [29]. A second study investigated QEEG in young adults who were born preterm and at extremely low birth weight, and who were free of neurosensory impairments and psychiatric disorders in adulthood [9]. This study found increased power in the slow (delta and theta) and decreased power in the fast (alpha and beta) frequency bands in preterm-born individuals, compared to term-born controls with an average birth weight of 3395 g, during a resting-state condition [9]. While it is now possible to study survivors of preterm birth due to major advances in neonatal care over the past few decades [3], the cortical activation patterns of preterm-born adolescents remain to be assessed. In addition, no study to date has directly compared cortical activation between preterm-born individuals and individuals with ADHD.

Research examining oscillatory patterns during cognitive task performance in ADHD has yielded inconsistent results. While some studies have reported elevated alpha [30, 31] and theta power [32] in individuals with ADHD compared to controls, others have reported no differences in EEG power between controls and individuals with ADHD during a continuous performance test (CPT) [13, 22, 33]. This lack of significant differences in EEG spectral power between controls and individuals with ADHD during the CPT was likely driven by the absence of rest-to-task transition effects in the ADHD group (i.e. no changes in spectral power from resting-state to cognitive task) in at least two of the studies reviewed above [13, 22]. To date, no studies have examined the QEEG profile of preterm-born individuals during cognitive task performance. Our previous direct comparisons across preterm-born adolescents and term-born adolescents with ADHD on event-related potential (ERP) measures associated with attentional and inhibitory processing from a cued continuous performance test (CPT-OX) showed impairments in response preparation (CNV), executive response control (Go-P3) and response inhibition (NoGo-P3) in preterm-born adolescents [34]. While the response preparation and response inhibition impairments found in preterm-born adolescents overlap with those found in term-born adolescents with ADHD, the preterm group also shows unique impairments in executive response control. Using the same ADHD and control group data included in this analysis, we previously reported findings on ADHD case–control differences in resting-state EEG spectral power at the beginning and end of a 1.5-h testing session [12]. We reported higher delta power in the ADHD group compared to controls at the beginning but not at the end of the testing session, as well as higher beta power in the ADHD group compared to controls at the end but not at the beginning of the testing session.

The aim of the present study was to test whether QEEG measures identify differences or similarities between preterm-born adolescents, term-born adolescents with ADHD and control adolescents during a resting-state condition (eyes open, EO) and a cognitive task condition (CPT-OX). This could inform us on impairments in brain function that may underlie symptomatic and cognitive similarities between ADHD and preterm birth. Understanding these impairments may help us to elucidate the risk pathways from preterm birth to ADHD, which currently remain poorly understood. Here, we compare new data obtained from preterm-born adolescents to data previously obtained from term-born ADHD and control participants during rest [12], and to data obtained from the same ADHD and control participants during the CPT-OX, which have not previously been investigated. As part of this study, we examine how EEG patterns change in relation to recording condition (resting vs. cognitive task) to investigate task-related modulation of EEG spectral power in preterm-born adolescents and adolescents with ADHD. No formal predictions were made for EEG spectral power during rest and task condition in the preterm group and in comparison with the ADHD group, owing to lack of evidence and inconsistency within the literature [9, 29].

## Method

### Measures

#### The Diagnostic Interview for ADHD in adults (DIVA)

The DIVA [35] is a semi-structured interview designed to evaluate the DSM-IV criteria for both adult and childhood ADHD symptoms and impairment. It consists of 18 items used to define the DSM-IV symptom criteria for ADHD. Each item is scored affirmatively if the behavioural symptom was present *often* within the past 6 months.

#### The Barkley Functional Impairment Scale (BFIS)

The BFIS [36] is a 10-item scale used to assess the levels of functional impairments commonly associated with ADHD symptoms in five areas of everyday life: family/relationship; work/education; social interaction; leisure activities; and management of daily responsibilities.

In the preterm and ADHD groups, ADHD was assessed using parental ADHD symptom ratings on the DIVA and the BFIS for all participants, for consistency. If participants were on stimulant medication, parents were instructed to consider their children’s ADHD symptoms off medication. A research diagnosis of ADHD was made if participants scored ≥6 on either the inattention or hyperactivity-impulsivity subscales of the DIVA and if they received ≥2 positive scores on ≥2 areas of impairment on the BFIS. In the control group, ADHD was assessed using parental ADHD symptom ratings on the BFIS for all participants, for consistency. Control participants were excluded from the analysis if they scored ≥2 on ≥2 areas of impairment on the BFIS.

#### IQ

The vocabulary and block design subtests of the Wechsler Abbreviated Scale of Intelligence-First Edition (WASI-I) [37] were administered to all participants to derive estimates of IQ.

#### Cued continuous performance test

The CPT-OX is a cued Go/NoGo task that probes attention, preparation and response inhibition. The task consisted of 400 black letter arrays, made up of a centre letter and incompatible flankers on each side to increase difficulty. The presented arrays included the cue letter ‘O’, the target letter ‘X’ as well as the distractors ‘H’, ‘B’, ‘C’, ‘D’, ‘E’, ‘F’, ‘G’, ‘J’ and ‘L’. Cue and target letters (‘O’ and ‘X’, respectively) were flanked by incompatible letters (‘XOX’ and ‘OXO’, respectively). Participants were instructed to ignore the flanking letters and respond as quickly as possible to cue-target sequences (‘O’–‘X’). 80 cues (‘XOX’) were followed by the target (‘OXO’) in 40 trials (Go condition), and by neutral distractors in the remainder of trials (NoGo condition). On 40 trials, the target letter ‘X’ was not preceded by a cue ‘O’ and had to be ignored. Letters were presented every 1.65 s for 150 ms in a pseudo-randomised order. Ten practice trials preceded the main task and were repeated, if required, to ensure participant comprehension. Participants were instructed to respond only to Cue-Go sequences by pressing a button as quickly as possible with the index finger of their preferred hand. Participants were further asked to withhold the response in the presence of a NoGo stimulus, in the presence of a Go stimulus not preceded by a cue, or in the presence of any other irrelevant letters. Task duration was 11 min.

Cognitive-performance measures obtained from the CPT-OX include mean reaction time (MRT in milliseconds), RTV (standard deviation of target reaction time), omission errors (OE; non-responses to Go trials) and commission errors (CE; responses to Cue, NoGo or distractor stimuli). MRT and RTV were obtained from correct Go trials.

### Sample

The sample consisted of 194 preterm-born participants, 93 ADHD participants and 166 controls. The groups differed significantly in terms of age, IQ, gender distribution, GA and ADHD symptom scores (Table 1). The ADHD group showed significantly higher ADHD symptoms than both the preterm (*t*_178_ = −16.55, *p* < 0.001) and control (*t*_134_ = 20.06, *p* < 0.001) groups. The preterm group further demonstrated significantly higher ADHD symptoms than the control group (*t*_213_ = 4.71, *p* < 0.001). 4% of the preterm-born participants and 47% of the ADHD participants were being treated with stimulant medication. A 48-h ADHD medication-free period was required prior to assessments. Written informed consent was obtained following procedures approved by the London–Surrey Borders Research Ethics Committee (09/H0806/58) and the National Research Ethics Service Committee London–Bromley (13/LO/0068).

**Table 1:** Descriptive statistics

	ADHD (*n* = 69)	Preterm (*n* = 186)	Control (*n* = 135)	Statistic	*df*	*p* value
GA in weeks (SD)	39.9 (1.4)	33.0 (3.0)	39.9 (1.3)	*t* = −23.0	253	<0.001
GA range in weeks	37–42	24–36	37–43	–	–	–
IQ (SD)	97.7 (13.8)	104.7 (12.3)	110.4 (12.2)	*t* = −3.2	253	0.002
Age (SD)	18.5 (3.0)	14.9 (1.9)	17.8 (2.1)	*t* = −12.0	253	<0.001
Age range	12.7–25.9	11.0–20.0	11.9–21.6	–	–	–
Males %	88.4	54.3	75.6	*t* = 4.6	253	<0.001
Conners’ parent-rated ADHD symptom score (SD)	35.8 (10.6)	11.2 (9.4)	7.0 (5.6)	*t* = 1.97	253	0.050
BFIS score (SD)	16.4 (5.4)	3.7 (4.1)	2.1 (2.5)	*t* = −2.23	253	0.027

*BFIS* Barkley Functional Impairment Scale

Exclusion criteria for all groups were IQ <70, general learning difficulties, cerebral palsy or any other medical conditions that affects motor co-ordination including epilepsy, as well as brain disorders and any genetic or medical disorder that might mimic ADHD. In addition, preterm birth was an exclusion criterion in the ADHD and control groups, because this study aimed to establish whether the cognitive impairments associated with preterm birth reflect identical neurophysiological impairments in term-born individuals with ADHD.

The preterm group was recruited from secondary schools in Southeast England. All preterm participants had one full sibling available for ascertainment, and were born before 37 weeks’ gestation. Siblings of preterm-born individuals were included in the preterm group if they were also born preterm to maximise the number of participants in the preterm group. Term-born siblings of preterm-born individuals were not included in this analysis. Ethnicities of the preterm-born participants included White European (84.6%), British Asian (3.7%), Mixed-White and Black Caribbean (2.1%), Mixed-White and British Asian (1.6%), Indian (1.1%), Mixed-White and Indian (1.1%), Black Caribbean (0.5%), Mixed-Black and British Asian (0.5%) and other (2.7%). Seven individuals from the preterm sample were excluded because medical birth records could not corroborate preterm status [gestational age (GA) ≥37 weeks]. One individual was excluded because of IQ <70. Eight preterm-born individuals met diagnostic criteria for a research diagnosis of ADHD. Since here preterm birth is investigated as a potential risk factor for ADHD, preterm-born individuals who demonstrated high levels of ADHD symptoms were not excluded from the analysis (for an analysis without preterm-born individuals who met a research diagnosis for ADHD, see Supplementary Material I).

ADHD and control sibling pairs, who had taken part in our previous research [38], were invited to take part in a follow-up study [39]. While ADHD–control differences for this sample have been reported previously in a study investigating the influence of recording context on EEG spectral power [12], here the ADHD and control groups are compared to a group of preterm-born adolescents. All participants were of White European descent and had one full sibling available for ascertainment. Participants with ADHD and their siblings were included in the ADHD group if they had a clinical diagnosis of DSM-IV combined-type ADHD during childhood and met DSM-IV criteria for any ADHD subtype at follow-up. Siblings of individuals with ADHD who did not meet DSM-IV criteria for any ADHD subtype at follow-up were not included in this analysis. The control group was initially recruited from primary (ages 6–11 years) and secondary (ages 12–18 years) schools in the UK, aiming for an age- and sex-match with the ADHD sample. Control individuals and their siblings were included in the control group if they did not meet DSM-IV criteria for any ADHD subtype either in childhood or at follow-up.

At follow-up, six participants from the ADHD–sibling pair sample were excluded from the group analyses because of missing parent ratings of clinical impairment. Therefore, their current ADHD status could not be determined. Two additional participants from the ADHD–sibling pair sample were excluded because of drowsiness during the cognitive task. Two participants with childhood ADHD, who did not meet ADHD symptom criteria but met clinical levels of impairment at follow-up, were excluded to minimise heterogeneity in the ADHD sample. Six control participants were removed from the analyses for meeting DSM-IV ADHD criteria based on the parent-rated Barkley Informant Rating Scale [36]. In addition to these exclusions, which are identical to our previous analysis [39], we also excluded six participants from the ADHD–sibling pair sample, who were born preterm, as well as 1 individual from the ADHD–sibling pair sample and 25 participants from the control–sibling pair sample, who provided no information on GA.

The final sample consisted of 186 preterm-born participants (41 sibling pairs, 104 singletons), 69 ADHD participants (4 sibling pairs, 61 singletons) and 135 controls (61 sibling pairs, 13 singletons).

### Procedure

Participants completed the cognitive-EEG assessments, including an IQ test and clinical interviews, in a single 4.5 h session. Participants completed a 3-min eyes-open resting-state condition (EO) as well as a 3-min EC resting-state condition prior to performing on a cued CPT (CPT-OX) [40]. QEEG differences between EO and CPT-OX are analysed here since EO has been suggested to provide a more appropriate baseline than EC for tasks involving visual processing [41].

### Electrophysiological recording

The EEG was recorded from a 62-channel direct-current-coupled recording system (extended 10–20 montage), using a 500-Hz sampling-rate and impedances under 10 kΩ. FCz and AFz were the recording reference and the ground electrodes, respectively. The electro-oculograms were recorded from electrodes above and below the left eye and at the outer canthi. Participants were seated on a height-adjustable chair in a dimly lit video-monitored testing cubicle. Stimuli were presented on a computer monitor at a distance of approximately 120 cm, using the Presentation software package (http://www.neurobs.com). EEG data were analysed using Brain Vision Analyzer 2.0 (Brain Products, Germany). Researchers were blind to group status during EEG pre-processing and analysis. Raw EEG recordings were down-sampled to 256 Hz, re-referenced to the average of all electrodes and digitally filtered using Butterworth band-pass filters (0.1–30 Hz, 24 dB/oct). All trials were also visually inspected for electrical artefacts (due to electrical noise in the EEG recording) or obvious movement, and sections of data containing artefacts were removed manually. Ocular artefacts, corresponding to blink-related and vertical and horizontal eye movements, were identified using the infomax independent component analysis (ICA) algorithm [42]. The ICA algorithm [42] allows for removal of activity associated with ocular artefacts by back-projection of all but this activity. Sections of data with remaining artefacts exceeding ±100 μV in any channel or with a voltage step greater than 50 μV were automatically rejected. Artefact-free data were segmented into 2-s epochs and power spectra were computed using a Fast Fourier Transform (FFT) with a 10% Hanning window. Only trials with correct responses (Go) or correctly rejected trials (NoGo and Cue), and which contained at least 20 artefact-free segments, were included.

**Fig. 1:**
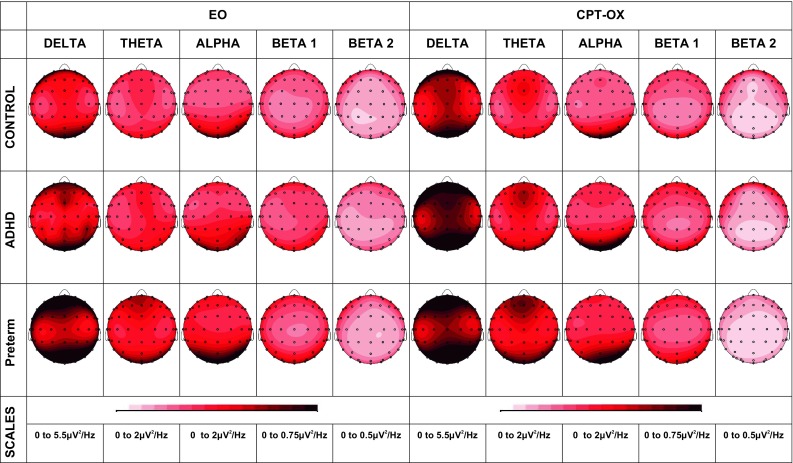
Topographic maps showing scalp recorded power density in delta, theta, alpha, beta 1 and beta 2 bands for resting-state (EO) and task (CPT-OX) conditions

Analyses focused on absolute delta (0.5–3.5 Hz), theta (3.5–7.5 Hz), alpha (7.5–12.5 Hz), beta 1 (12.5–18.5 Hz) and beta 2 (18.5–30 Hz) frequency band [11–14] differences between preterm, ADHD and control groups. All data were log transformed (lg) to normalise the data. The normal distribution of log-transformed data was confirmed using a Shapiro–Wilk test. In line with previous studies [22, 33], absolute EEG power (μV^2^) within each frequency band was averaged across frontal (Fz, F1, F2, F3, F4, F5, F6, F7, F8), central (Cz, C1, C2, C3, C4, C5, C6) and parietal (Pz, P3, P4, P7, P8) regions from individual scalp electrodes to reduce the number of statistical comparisons (see Fig. 1 for topographic maps showing scalp recorded power density in the frequency bands).

### Statistical analysis

Data were analysed using random intercept models in Stata, to control for non-independence of the data, i.e. data coming from siblings of one family, in a repeated-measures design, using a ‘robust cluster’ command to estimate standard errors [43]. Regression-based corrections for age were applied to raw scores and residual scores were analysed. All analyses controlled for gender. All analyses were re-run with IQ as a covariate. To investigate how neurophysiological impairments relate to ADHD symptoms in the preterm group, correlations were run between delta power and DIVA ADHD symptom scores. The functional significance of delta power was investigated by running correlations between cognitive performance measures from the CPT-OX and delta power during CPT-OX as well as change in delta power from EO to CPT-OX. Effect size (Cohen’s *d*), which was calculated using the difference in the means divided by the pooled standard deviation, is also reported [44]. According to Cohen [44], *d* = 0.20 constitutes a small effect, *d* = 0.50 a medium effect and *d* = 0.80 a large effect.

## Results

**Fig. 2:**
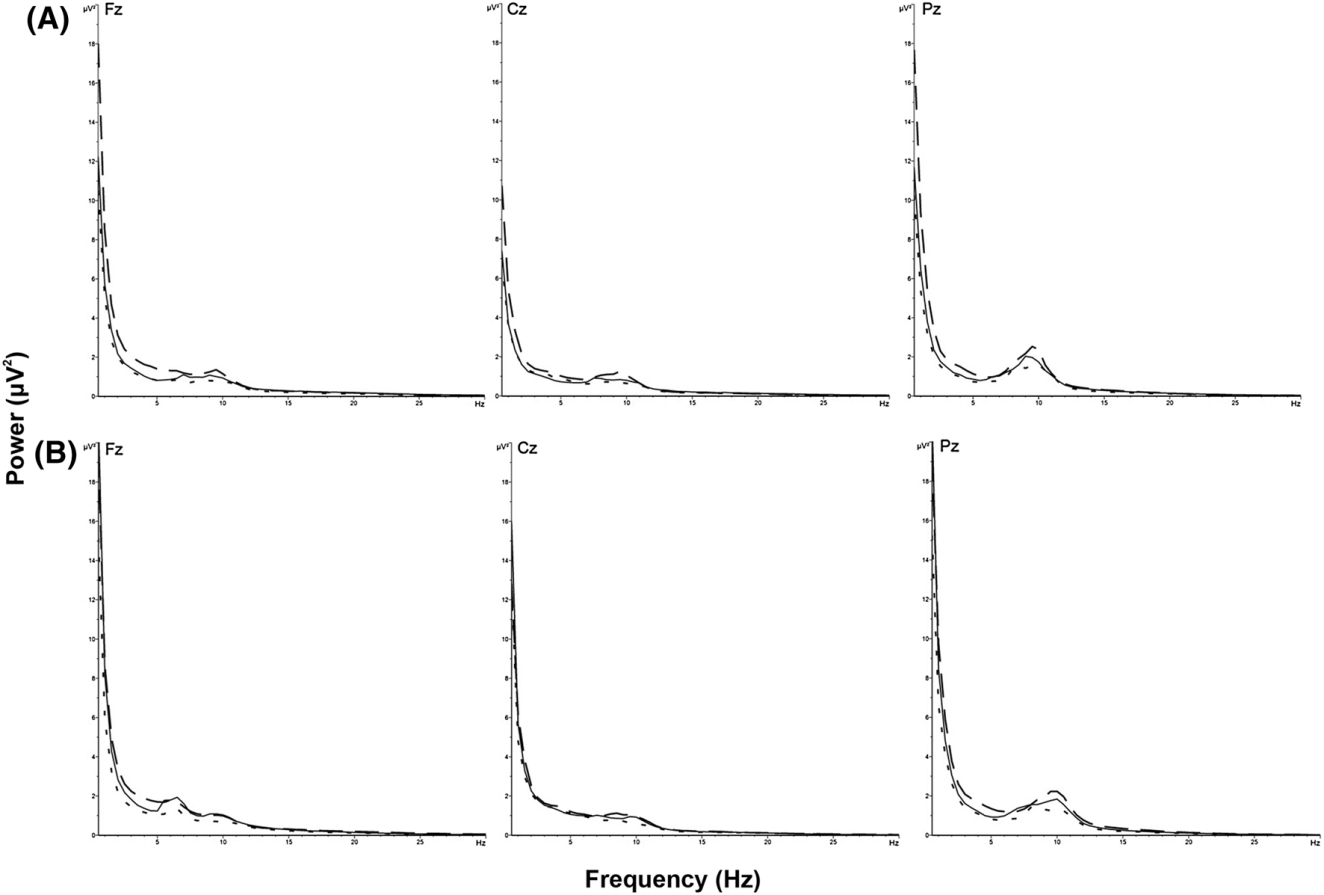
Power spectra for the preterm-born (*dashed line*), ADHD (*solid line*) and control (*dotted line*) groups during the **a** resting-state (EO) and **b** task (CPT-OX) conditions. *Plots* represent raw absolute power at Fz, Cz and Pz

The random intercept model indicated no significant main effects of group for absolute alpha (*z* = −0.87, *p* = 0.382), beta 1 (*z* = −0.12, *p* = 0.904), beta 2 (*z* = 0.16, *p* = 0.869), theta (*z* = 0.62, *p* = 0.535) or delta (*z* = −1.81, *p* = 0.070) power (Fig. 2). A significant main effect of group emerged for absolute delta power (*z* = −1.97, *p* = 0.049) when IQ was included as a covariate. No significant main effects of group emerged for absolute alpha (*z* = 1.27, *p* = 0.205), beta 1 (*z* = 0.58, *p* = 0.560), beta 2 (0.05, *p* = 0.958), or theta (*z* = 0.81, *p* = 0.420) power when IQ was included as a covariate.

Significant main effects of condition arose for absolute beta 2 (*z* = −2.39, *p* = 0.017), theta (*z* = 10.05, *p* < 0.001) and delta (*z* = 7.00, *p* < 0.001) power. No significant main effects of condition were found for absolute alpha (*z* = −1.26, *p* = 0.207) and beta 1 (*z* = 0.37, *p* = 0.712) power. Significant main effects of condition arose for absolute beta 2 (*z* = −2.83, *p* = 0.005), theta (*z* = 9.76, *p* < 0.001) and delta (*z* = 7.77, *p* < 0.001) power when IQ was included as a covariate. No significant main effects of condition were found for absolute alpha (*z* = −0.90, *p* = 0.370) and beta 1 (*z* = 0.58, *p* = 0.560) power when IQ was included as a covariate.

Significant main effects of site were found for alpha (*z* = 19.28, *p* < 0.001), beta 1 (*z* = 10.96, *p* < 0.001), beta 2 (*z* = −12.74, *p* < 0.001) and theta (*z* = 3.80, *p* < 0.001) power. The random intercept model further indicated no significant main effects of site for absolute delta power (*z* = −1.46, *p* = 0.144). Significant main effects of site were found for alpha (*z* = 14.78, *p* < 0.001), beta 1 (*z* = 10.82, *p* < 0.001), beta 2 (*z* = −13.56, *p* < 0.001) and theta (*z* = 3.68, *p* < 0.001) power when IQ was included as a covariate. The random intercept model further indicated no significant main effects of site for absolute delta power (*z* = −1.69, *p* = 0.091) when IQ was included as a covariate.

**Fig. 3:**
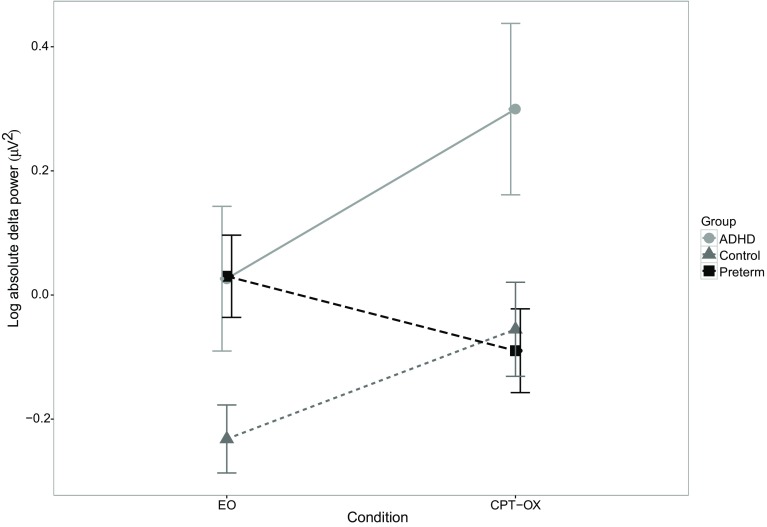
Mean absolute delta power across resting-state (EO) and task (CPT-OX) condition in the preterm-born adolescents (*dashed line with square marker*), ADHD (*solid line with round marker*) and control groups (*dotted line with triangular marker*). *Error bars* represent 95% confidence intervals

The random intercept model yielded a significant group-by-condition interaction for absolute delta power (*z* = −6.71, *p* < 0.001) (Fig. 3). No significant group-by-condition interactions were found for absolute alpha (*z* = −0.88, *p* = 0.380), beta 1 (*z* = −1.13, *p* = 0.260), beta 2 (*z* = −0.75, *p* = 0.456) or theta (*z* = −1.86, *p* = 0.064) power. When IQ was included as a covariate, the random intercept model yielded a significant group-by-condition interaction for absolute delta power (*z* = −6.70, *p* < 0.001), but no significant group-by-condition interactions were found for absolute alpha (*z* = −0.87, *p* = 0.382), beta 1 (*z* = −1.12, *p* = 0.261), beta 2 (*z* = −0.74, *p* = 0.458) or theta (*z* = −1.85, *p* = 0.065) power.

A significant group-by-site interaction was found for absolute beta 1 power (*z* = 2.35, *p* = 0.019) and a significant group-by-condition-by-site interaction was found for absolute delta power (*z* = −2.37, *p* = 0.018). No other significant interactions were found. When IQ was included as a covariate, a significant group-by-site interaction was also found for absolute beta 1 power (*z* = 2.36, *p* = 0.018) and a significant group-by-condition-by-site interaction was found for absolute delta power (*z* = −2.38, *p* = 0.018). No other significant interactions were found when IQ was included as a covariate.

Post‐hoc regression analyses revealed significantly higher delta power during EO in the preterm group compared to the control group (*t* = 3.47, *p* = 0.001), with moderate effect size (*d* = 0.31), but not compared to the ADHD group (*t* = 0.84, *p* = 0.400). As previously reported [12], significantly higher delta power during EO was also found in the ADHD group compared to controls (*t* = 4.56, *p* < 0.001), with moderate effect size (*d* = 0.32). During CPT-OX, the preterm and control groups did not differ significantly with regard to delta power. However, the ADHD group showed significantly higher delta power compared to both the control (*t* = 4.16, *p* < 0.001) and preterm (*t* = −4.52, *p* < 0.001) groups during CPT-OX, with moderate-to-large effect sizes (*d* = 0.57 and *d* = 0.77, respectively). In the ADHD group, increased delta power during CPT-OX was significantly correlated with poorer cognitive performance in the CPT-OX, namely with increased MRT (*r* = 0.28, *p* = 0.030), RTV (*r* = 0.37, *p* = 0.004), number of OE (*r* = 0.32, *p* = 0.044) and number of CE (*r* = 0.28, *p* = 0.047). In the preterm group, delta power during CPT-OX was not significantly correlated with CPT-OX MRT (*r* = 0.004, *p* = 0.956), RTV (*r* = 0.07, *p* = 0.347), OE (*r* = 0.02, *p* = 0.882) or CE (*r* = −0.12, *p* = 0.174). In the control group, delta power during CPT-OX was not significantly correlated with CPT-OX performance timing on MRT (*r* = −0.11, *p* = 0.236) or RTV (*r* = 0.06, *p* = 0.506), but delta power increases showed a significant association with poorer CPT-OX performance on accuracy measures OE (*r* = 0.43, *p* = 0.005) and CE (*r* = 0.38, *p* = 0.0003). When IQ was included as a covariate, the preterm group also demonstrated higher delta power during EO compared to the control group (*t* = 3.19, *p* = 0.002), with moderate effect size (*d* = 0.30), but not compared to the ADHD group (*t* = 1.00, *p* = 0.319). As previously reported [12], when controlling for IQ higher group differences between the ADHD and control group in delta power during EO weakened to non-significance (*t* = 1.44, *p* = 0.151), with small effect size (*d* = 0.19).

Post‐hoc regression analyses further demonstrated a significant decrease in delta from EO to CPT-OX in the preterm group (*t* = −3.30, *p* = 0.001), as well as a significant increase in delta power from EO to CPT-OX in the ADHD group (*t* = 3.79, *p* < 0.001). No significant change from EO to CPT-OX was found in the control group (*t* = 1.61, *p* = 0.112) (Fig. 3). In the preterm and ADHD groups, change in delta power from EO to CPT-OX was not significantly correlated with the CPT-OX performance measures MRT (*r* = −0.01, *p* = 0.909; *r* = −0.18, *p* = 0.160, respectively), RTV (*r* = 0.01, *p* = 0.936; *r* = 0.12, *p* = 0.347, respectively), OE (*r* = −0.09, *p* = 0.447; *r* = 0.02, *p* = 0.904, respectively) or CE (*r* = 0.03, *p* = 0.740; *r* = −0.08, *p* = 0.562). Since no significant change from EO to CPT-OX was found in the control group, correlations between change in delta power and cognitive-performance measures were not run.

DIVA ADHD symptom scores in the preterm group were significantly positively correlated with delta power during EO (*r* = 0.17, *p* = 0.028), but not CPT-OX (*r* = 0.11, *p* = 0.25). When IQ was included as a covariate, DIVA ADHD symptom scores in the preterm group were significantly positively correlated with delta power during EO (*r* = 0.17, *p* = 0.029), but not CPT-OX (*r* = 0.10, *p* = 0.21).

Excluding the eight preterm-born individuals meeting diagnostic criteria for a research diagnosis of ADHD from the analyses did not alter the results (supplementary material I; see Table S1 for a comparison of gestational age, IQ, age and gender distribution between the preterm individuals with and without a research diagnosis ADHD). Excluding females from the analyses did not alter the results either (supplementary material II).

## Discussion

In this study investigating the relationship of EEG indices of cortical activity in preterm-born adolescents, term-born adolescents with ADHD and term-born controls, the preterm group showed higher absolute delta power compared to controls during the resting-state condition, as observed in the ADHD group [12]. No significant differences emerged between the preterm and ADHD groups at rest. Concurrently, parent-rated ADHD symptoms in the preterm group were significantly positively correlated with delta power during rest. Furthermore, no significant differences in delta power arose between the preterm and control groups during CPT-OX, whereas the ADHD group displayed significantly higher delta power during CPT-OX compared to both the preterm and control groups. These findings provide evidence for commonalities and differences in oscillation patterns between preterm-born adolescents and adolescents with ADHD in the delta range. Increased delta power during rest may be a potential general marker of brain trauma or pathology.

Increased delta power during resting state was observed in the preterm group, similar to the ADHD group [12], with both preterm and ADHD groups significantly different from controls. While previous research has shown an elevation of delta power during resting-state conditions independently in individuals with ADHD [15–18] and in individuals born preterm [9], compared to controls, the present study is the first direct comparison between the groups using identical methods. These findings suggest commonalities in delta power between preterm-born adolescents and term-born adolescents with ADHD. In line with these findings, parent-rated ADHD symptoms in the preterm group showed a significant positive relationship with delta power during rest. It is not fully understood what increased delta power during resting-state conditions represents in preterm-born individuals and individuals with ADHD. Yet, as increased delta power in resting EEG has been reported in a wide range of psychopathologies, including autism spectrum disorder (ASD) [45], depression [46] and schizophrenia [47], the lack of specificity may suggest that increased delta power during resting-state is a potential general marker of brain trauma or pathology. It is conceivable that preterm birth may result in trauma to some of the brain networks associated with ADHD, as well as networks associated with additional impairments, since the late third trimester (32–40 weeks’ gestation) serves as a critical period to lay the foundation of vital brain networks [5]. This idea is supported by research suggesting that, as well as being a risk factor for ADHD, preterm birth presents a risk factor for other psychiatric disorders, such as ASD, depression and schizophrenia [48]. Routine psychiatric screening to facilitate early psychological referral is, therefore, likely to be valuable in this at-risk population.

In contrast, no significant differences in delta power arose between the preterm and control groups during CPT-OX, whereas the ADHD group demonstrated significantly higher delta power compared to the preterm group during CPT-OX. Concurrently, no significant correlation between parent-rated ADHD symptoms and delta power were found in the preterm group during CPT-OX. The association of increased CPT-OX delta power with poorer CPT-OX task performance, particularly in the ADHD group, suggests that delta power not only reflects impairment during the resting state in the preterm group, but also the impairment of the ADHD group during the attentional challenge during CPT-OX task performance, and thereby supports the finding of partly distinct profiles of impairment in these two groups. While delta power significantly decreased in preterm-born individuals from the rest condition to the task condition, the ADHD group showed an increase in delta power and no significant change in delta power from EO to CPT-OX was seen in the control group. The findings, therefore, suggest differences in brain function between preterm-born individuals and individuals with ADHD during CPT-OX, as well as differences in task-dependent modulation of absolute delta power. Pertinently, delta power has been suggested to play a role in the modulation of the default mode network (DMN) [49], which is typically activated during resting-state conditions and deactivated during task performance [50]. Previous research suggested enhanced suppression of DMN regions during higher workload in very preterm-born adolescents, compared to controls, in order to maintain adequate task performance with increasing attentional demands [51]. Abnormalities in the DMN during rest and inadequate attenuation during task performance have also been demonstrated for individuals with ADHD [52, 53]. The increase in absolute delta power in our ADHD group could, therefore, indicate inadequate attenuation of the DMN. Future research is needed to examine the task-dependent deactivation of the DMN in preterm-born adolescents from resting-state to CPT-OX further to elucidate whether preterm-born individuals compensate for higher delta power during resting-state with a stronger task-dependent deactivation of the DMN.

Moreover, power in the delta band was significantly higher in the ADHD group compared to controls during both the resting-state [12] and the task conditions. These results do not replicate previous research in men [22] and women [13] with adult ADHD, which reported higher theta power in the ADHD group compared to controls during EO, and no significant differences in theta power between controls and individuals with ADHD during the CPT-OX. In addition, no change in EEG spectral power from resting-state to cognitive task was found in the ADHD group in these studies [22]. While it is conceivable that heterogeneity in the ADHD samples with regard to ADHD subtype [20], medication status [54] and comorbidities [55] could have resulted in inconsistencies between studies, it seems more likely that differences in sample ages may have contributed to the discrepancies between the current findings in adolescents and previous findings in adults with ADHD [13, 22]. The observation that EEG spectral power in all frequency bands is affected by age has also been shown in other studies of individuals with ADHD [21, 25, 26]. Research further indicates the possibility that developmental trajectories of EEG spectral power in individuals with ADHD may not be linear throughout the lifespan. In one study children with ADHD displayed higher delta power than controls while no such differences were found in adults with ADHD compared to controls [19]. Yet, longitudinal studies are needed to fully elucidate the trajectory of EEG spectral power development in ADHD and to examine these discrepancies in rest-to-task transition effects in ADHD.

A few limitations should be considered along with the present results. First, we examined cortical activation for fixed frequency bands. While some researchers have advocated abandoning fixed frequency bands [56], the grouping of these frequencies into the particular bands has occurred historically and makes comparison between studies easier. Moreover, QEEG provides averaged measures of cortical activation across the resting-state and the cognitive task condition. To explore the various cognitive processes underlying cortical activation further, future research may employ time–frequency analysis. For finer resolution and increased signal to noise ratio, source-based analyses, such as ICA, could also be applied. Second, the preterm group is younger, on average, than the ADHD and control groups. While the possibility of age effects on QEEG measures cannot be precluded, regression-based corrections for age were applied to raw scores and residual scores were analysed. Future research is needed to examine age-related changes in spectral power in preterm-born individuals to establish whether alterations in resting-state EEG might index delayed brain maturation in individuals born preterm.

Our results provide some of the first QEEG evidence for commonalities in oscillation patterns in the delta range during rest, but differences with regard to delta power during CPT-OX and rest-to-task transition effects, between preterm-born adolescents and term-born adolescents with ADHD. Overall, our results suggest that preterm birth may present a risk factor for both ADHD and additional impairments. This vulnerable population may, therefore, benefit from routine psychiatric screening to facilitate early psychological referral. As this is one of the first studies to directly compare preterm-born adolescents to term-born adolescents with ADHD and term-born controls on measures of cortical activation, these findings require replication in larger-scale studies. Future studies should build on these results by also investigating the relationship between preterm birth and ADHD and associated neurocognitive impairments at various developmental stages such as in very early childhood during the development of neural networks and in mid-childhood when ADHD is typically diagnosed.

## Electronic supplementary material

Below is the link to the electronic supplementary material.

Supplementary material 1 (DOCX 16 kb)

Supplementary material 2 (DOCX 25 kb)

Supplementary material 3 (DOCX 21 kb)
