# Supplementary material for: Altered EEG spectral power during rest and cognitive performance: a comparison of preterm-born adolescents to adolescents with ADHD
Source: Eur Child Adolesc Psychiatry. 2017 Jun 2;26(12):1511–22. doi: 10.1007/s00787-017-1010-2 (PMC5600884; doi:10.1007/s00787-017-1010-2)
Supplement: Supplementary file 1 — Supplementary material 1 (DOCX 16 kb) [file 787_2017_1010_MOESM1_ESM.docx]

|  | **Preterm-born individuals with ADHD** | **Preterm-born individuals without ADHD** | **z** | **p-value** |
| --- | --- | --- | --- | --- |
|  | n=13 | n=167 | - | - |
| **GA in weeks (SD)** | 33.8 (3.2) | 32.9 (3.0) | -0.81 | 0.418 |
| **GA range in weeks** | 27 - 36 | 24 - 36 | - | - |
| **IQ (SD)** | 95.7 (13.7) | 105.4 (12.0) | 3.1 | 0.002 |
| **Age (SD)** | 14.6 (1.5) | 15.0 (1.9) | 0.73 | 0.468 |
| **Age range** | 12.2-16.9 | 11.2-20.0 | - | - |
| **Males %** | 92.3 | 50.9 | 2.95 | 0.003 |
