# Supplementary material for: Altered EEG spectral power during rest and cognitive performance: a comparison of preterm-born adolescents to adolescents with ADHD
Source: Eur Child Adolesc Psychiatry. 2017 Jun 2;26(12):1511–22. doi: 10.1007/s00787-017-1010-2 (PMC5600884; doi:10.1007/s00787-017-1010-2)
Supplement: Supplementary file 2 — Supplementary material 2 (DOCX 25 kb) [file 787_2017_1010_MOESM2_ESM.docx]

## Supplementary material I – Results without the preterm-born individuals with a research diagnosis of ADHD

The subsample consisted of 69 ADHD participants, 178 preterm-born participants and 135 controls. Significant differences in GA, IQ, age and gender distribution were observed (Table S1). The ADHD group showed significantly higher Conners’ parent-rated ADHD symptoms and functional impairment scores on the Barkley Functional Impairment Scale than both the preterm (t=-17.65, df=105, p<0.001 and t=-19.30, df=105, p<0.001 respectively) and control groups (t=19.38, df=79, p<0.001 and t=17.13, df=79, p<0.001 respectively). The preterm group further demonstrated significantly higher Conners’ parent-rated ADHD symptom scores (t=2.63, df=136, p=0.009), but not functional impairment scores on the Barkley Functional Impairment Scale (t= 0.02, df=136, p= 0.985), compared to the control group.

**Results**

The random intercept model indicated no significant main effects of group for absolute alpha (z=0.73, p=0.468), beta 1 (z=0.20, p=0.842), beta 2 (z=0.02, p=0.987) or theta (z=0.10, p=0.918) power. A significant main effect of group emerged for absolute delta power (z=-2.12, p=0.034).

No significant main effects of condition were found for absolute alpha (z=0.37, p=0.715) and beta 1 (z=0.22, p=0.777) power. Significant main effects of condition arose for absolute beta 2 (z=-2.72, p=0.007), theta (z=9.81, p<0.001) and delta (z=7.99, p<0.001) power.

The random intercept model further indicated no significant main effects of site for absolute delta power (z=-1.66, p=0.098). Significant main effects of site were found for alpha (z=19.24, p<0.001), beta 1 (z=10.56, p<0.001), beta 2 (z=-13.13, p<0.001) and theta (z=3.78, p<0.001) power.

The random intercept model yielded a significant group-by-condition interaction for absolute delta power (z=-6.44, p<0.001) (Figure 2). No significant group-by-condition interactions were found for absolute alpha (z=-1.04, p=0.300), beta 1 (z=-1.13, p=0.259), beta 2 (z=-0.70, p=0.482) or theta (z=-1.77, p=0.076) power. Post‐hoc regression analyses revealed significantly higher delta power during EO in the preterm group compared to the control group (t=3.04, p=0.003), with small to moderate effect size (d=0.28), but not compared to the ADHD group (t=0.55, p=0.585). As previously reported (Kitsune et al., 2015), significantly higher delta power during EO was also found in the ADHD group compared to controls (t=4.56, p<0.001), with moderate effect size (d=0.32). During CPT-OX, the preterm and control groups did not differ significantly with regard to delta power (t=-0.71, p=0.477). However, the ADHD group showed significantly higher delta power compared to both the control (t=4.16, p<0.001) and preterm (t=-4.74, p<0.001) groups during CPT-OX, with moderate effect sizes (d=0.57 and d=0.57 respectively). Post‐hoc regression analyses further demonstrated a significant decrease in delta from EO to CPT-OX in the preterm group (t=-3.12, p=0.005), as well as a significant increase in delta power from EO to CPT-OX in the ADHD group (t=3.84, p<0.001). No significant change from EO to CPT-OX was found in the control group (t=1.24, p=0.220) (Figure 2). DIVA ADHD symptom scores in the preterm group were not significantly correlated with delta power during EO (r=0.04, p=0.60) or CPT-OX (r=-0.01, p=0.88).

**Theta/beta ratio (TBR)**

No significant main effect of group (z=-0.94, p=0.348), condition (z=-1.00, p=0.315) or site (z=0.05, p=0.963), and no significant group-by-condition interaction emerged for TBR (z=1.14, p=0.253).

Table S1. Descriptive statistics for the sample without the preterm-born individuals with a research diagnosis of ADHD.

|  | **ADHD** | **Preterm** | **Control** | **Statistic** | **p-value** |
| --- | --- | --- | --- | --- | --- |
|  | n=69 | n=178 | n=135 | - | - |
| **GA in weeks (SD)** | 39.9 (1.4) | 33.0 (2.9) | 39.9 (1.3) | t=-22.1 | <0.001 |
| **IQ (SD)** | 97.7 (13.8) | 105.1 (12.3) | 110.4 (12.2) | t=-2.9 | 0.004 |
| **Age (SD)** | 18.5 (3.0) | 15.1 (1.8) | 17.8 (2.1) | t=-11.1 | <0.001 |
| **Age range** | 12.7-25.9 | 12.0-20.0 | 11.9-21.6 | - | - |
| **Males %** | 88.4 | 54.0 | 75.6 | t=4.6 | <0.001 |
| **Conners parent rated ADHD symptom score (SD)** | 35.8 (10.6) | 10.7 (8.7) | 7.0 (5.6) | t=-1.1 | 0.294 |
| **BFIS score (SD)** | 16.4 (5.4) | 3.5 (3.7) | 2.1 (2.5) | t=-0.98 | 0.329 |
