# Supplementary material for: Altered EEG spectral power during rest and cognitive performance: a comparison of preterm-born adolescents to adolescents with ADHD
Source: Eur Child Adolesc Psychiatry. 2017 Jun 2;26(12):1511–22. doi: 10.1007/s00787-017-1010-2 (PMC5600884; doi:10.1007/s00787-017-1010-2)
Supplement: Supplementary file 3 — Supplementary material 3 (DOCX 21 kb) [file 787_2017_1010_MOESM3_ESM.docx]

**Supplementary material II – Analysis of male subsample**

The final age-matched subsample consisted of 100 preterm-born participants 58 term-born participants with ADHD and 95 term-born controls. Significant differences in age (z=-23.64, p<0.001), IQ (z=17.06, p<0.001) and GA (z=-57.06, p<0.001) were observed, similar to the entire sample.

**Results**

The random intercept model indicated no significant main effects of group for absolute alpha (z=1.85, p=0.065), beta 1 (z=0.64, p=0.524), beta 2 (z=-0.52, p=0.602), theta (z=1.80, p=0.071) or delta (z=0.80, p=0.425) power.

Significant main effects of condition arose for absolute beta 2 (z=-2.75, p=0.006), theta (z=10.31, p<0.001) and delta (z=8.93, p<0.001) power. No significant main effects of condition were found for absolute alpha (z=-0.85, p=0.397) and beta 1 (z=0.48, p=0.632) power.

The random intercept model yielded a significant group-by-condition interaction for absolute delta power (z=-5.40, p<0.001) (Figure 2). No significant group-by-condition interactions were found for absolute alpha (z=-0.17, p=0.867), beta 1 (z=-1.35, p=0.178), beta 2 (z=-1.10, p=0.272) or theta (z=-1.46, p=0.145) power.

Post‐hoc regression analyses revealed significantly higher delta power during EO in the preterm group compared to the control group (t=4.10, p<0.001), with moderate effect size (d=0.20), but not compared to the ADHD group (t=-1.56, p=0.122). Trend-level higher delta power during EO was also found in the ADHD group compared to controls (t=1.90, p=0.059), with moderate effect size (d=0.37). During CPT-OX, the preterm and control groups did not differ significantly with regard to delta power (t=-0.47, p=0.637). However, the ADHD group showed significantly higher delta power compared to both the control (t=3.83, p<0.001) and preterm (t=-3.93, p<0.001) groups during CPT-OX, with moderate-to-large effect sizes (d=0.53 and d=0.61 respectively). Post‐hoc regression analyses further demonstrated a significant decrease in delta from EO to CPT-OX in the preterm group (t=-3.12, p=0.003), as well as a significant increase in delta power from EO to CPT-OX in the ADHD (t=2.26, p=0.032) and control (t=2.10, p=0.039) groups. DIVA ADHD symptom scores in the preterm group were positively correlated with delta power during EO at trend-level (r=0.19, p=0.067). DIVA ADHD symptom scores in the preterm group were not significantly correlated with delta power during CPT-OX (r=0.05, p=0.65).
